# IL-10-Dependent Amelioration of Chronic Inflammatory Disease by Microdose Subcutaneous Delivery of a Prototypic Immunoregulatory Small Molecule

**DOI:** 10.3389/fimmu.2021.708955

**Published:** 2021-07-08

**Authors:** Jorge H. Tabares-Guevara, Julio C. Jaramillo, Laura Ospina-Quintero, Christian A. Piedrahíta-Ochoa, Natalia García-Valencia, David E. Bautista-Erazo, Erika Caro-Gómez, Camila Covián, Angello Retamal-Díaz, Luisa F. Duarte, Pablo A. González, Susan M. Bueno, Claudia A. Riedel, Alexis M. Kalergis, José R. Ramírez-Pineda

**Affiliations:** ^1^ Grupo Inmunomodulación (GIM), Instituto de Investigaciones Médicas, Facultad de Medicina, Corporación Académica para el Estudio de Patologías Tropicales (CAEPT), Universidad de Antioquia, Medellín, Colombia; ^2^ Millennium Institute on Immunology and Immunotherapy, Departamento de Genética Molecular y Microbiología, Facultad de Ciencias Biológicas, Pontificia Universidad Católica de Chile, Santiago, Chile; ^3^ Departamento de Ciencias Biológicas, Facultad de Ciencias de la Vida, Millennium Institute on Immunology and Immunotherapy, Universidad Andrés Bello, Santiago, Chile; ^4^ Departamento de Endocrinología, Escuela de Medicina, Facultad de Medicina, Pontificia Universidad Católica de Chile, Santiago, Chile

**Keywords:** Interleukin-10, atherosclerosis, experimental autoimmune encephalomyelitis, chronic inflammatory disease, immune regulation, curcumin

## Abstract

One of the interventional strategies to reestablish the immune effector/regulatory balance, that is typically altered in chronic inflammatory diseases (CID), is the reinforcement of endogenous immunomodulatory pathways as the one triggered by interleukin (IL)-10. In a recent work, we demonstrated that the subcutaneous (sc) administration of an IL-10/Treg-inducing small molecule-based formulation, using a repetitive microdose (REMID) treatment strategy to preferentially direct the effects to the regional immune system, delays the progression of atherosclerosis. Here we investigated whether the same approach using other IL-10-inducing small molecule, such as the safe, inexpensive, and widely available polyphenol curcumin, could induce a similar protective effect in two different CID models. We found that, in apolipoprotein E deficient mice, sc treatment with curcumin following the REMID strategy induced atheroprotection that was not consequence of its direct systemic lipid-modifying or antioxidant activity, but instead paralleled immunomodulatory effects, such as reduced proatherogenic IFNγ/TNFα-producing cells and increased atheroprotective FOXP3^+^ Tregs and IL-10-producing dendritic and B cells. Remarkably, when a similar strategy was used in the neuroinflammatory model of experimental autoimmune encephalomyelitis (EAE), significant clinical and histopathological protective effects were evidenced, and these were related to an improved effector/regulatory cytokine balance in restimulated splenocytes. The essential role of curcumin-induced IL-10 for neuroprotection was confirmed by the complete abrogation of the clinical effects in IL-10-deficient mice. Finally, the translational therapeutic prospection of this strategy was evidenced by the neuroprotection observed in mice starting the treatment one week after disease triggering. Collectively, results demonstrate the power of a simple natural IL-10-inducing small molecule to tackle chronic inflammation, when its classical systemic and direct pharmacological view is shifted towards the targeting of regional immune cells, in order to rationally harness its immunopharmacological potential. This shift implies that many well-known IL-10-inducing small molecules could be easily reformulated and repurposed to develop safe, innovative, and accessible immune-based interventions for CID.

## Introduction

Chronic non-communicable diseases including cardiometabolic, neurodegenerative, musculoskeletal, allergic, and autoimmune diseases are the major causes of human morbidity and mortality worldwide (1). Behavioral, nutritional, and environmental risk factors associated with modern life, as well as immunogenetic factors, might trigger primary or secondary innate and adaptive immune reactions able to promote low grade but sustained inflammatory responses, that mediate organ dysfunction and injury (2). Given the variety of organs and systems that could be affected, the management of chronic inflammatory diseases (CID) is complex and disease dependent. However, as a general rule, interventions are usually focused on mitigating or eliminating risk factors, improving the function of the affected organs, and reducing the inflammatory burden by using poorly specific immunosuppressive and/or anti-inflammatory drugs (3–6). Advances in understanding of the immunopathological mechanisms underlying these diseases have identified proinflammatory mediators amenable for highly specific targeting with biological agents, such as anti-TNFα, anti-IL-6, or anti-IL-1β monoclonal antibodies (mAbs). However, although inflammatory responses are substantially impacted by these agents, immunosuppression and high financial costs remain as major limitations (7–9). The recent realization that the rupture of the delicate balance between effector and regulatory immune mechanisms characterizing CID (2, 3, 10) are not the sole result of an exaggerated proinflammatory response, but also of an impaired anti-inflammation/resolution response, has permitted to propose the reinforcement of endogenous regulatory mechanisms as an alternative interventional approach. From the leukocyte types that exert inflammation resolution-related actions, regulatory T cells (Tregs) have emerged as prominent players. Several strategies that restore Tregs numbers and activities have been proposed, including cell transfer therapy (with Tregs or tolerogenic dendritic cells, tolDC), biological agents such as rIL-2, or sophisticated particle-based delivery systems (11–15). Most of these approaches, however, are still in early development, and the safety concerns, high costs and technical complexity are limitations that hamper their possible implementation to routine widespread clinical practice (7–9, 16, 17).

Abundant scientific evidence supports the role of the regulatory cytokine IL-10 as a master player among the soluble factors that promote tolerance and homeostasis [(18) and references therein]. Proinflammatory effector T cells typically linked to CID (such as Th1 or Th17) are known to co-express IL-10 in addition to their signature Th cytokines, as a feedback regulatory loop to prevent an excessive response. Moreover, several major types of regulatory innate and adaptive leukocytes, such as tolDC, Myeloid-derived suppressor cells (MDSC), Tregs, or regulatory B cells (Bregs), execute tolerogenic programs through IL-10 production, which in turn, mediates infectious tolerance, a key immune process that prevents immunopathology (18, 19). Furthermore, many CID are characterized by IL-10 production impairment (18), and responsiveness to some treatments correlate with the re-establishment of IL-10 production (20). On this basis, and findings from preclinical CID models, systemic administration of rIL-10 has been tested in human clinical trials, with poor safety/efficacy outcomes as a consequence of the pleiotropism, complexity and paradoxical proinflammatory actions of this cytokine. The current view is that to harness the power of this cytokine for immune-related diseases, novel strategies, beyond the simplistic systemic rIL-10 supply, are required (18, 21, 22). An alternative approach that might offer advantages such as low cost and easy preclinical and clinical development is the exploitation of known IL-10-inducing small molecules. A number of synthetic and natural small molecules (such as dexamethasone, Dexa, and vitamin D, VitD) have been reported to act as IL-10 inducers *in vitro* and *in vivo* (23–27), and investigations that explore different formulations, delivery systems, and route/dose/scheme of administration could be of particular interest to develop realistic forms of immune interventions for CID. Based on the synergistic activity of VitD and Dexa to induce IL-10 and promote Tregs (28, 29), we recently postulated and demonstrated that a subcutaneous (sc) repetitive microdose delivery (REMID) strategy of a combined VitD/Dexa formulation increased the number of IL-10-producing leukocytes, reduced the Th1 proinflammatory response and attenuated the progression of atherosclerotic lesions (30), the major underlying cause of cardiovascular disease (CVD). These results constitute the preclinical proof of concept for a simple inexpensive hydrophobic small molecule-based formulation that can be repurposed to promote IL-10-producing cells *in vivo* and, therefore, as a potential safe, effective, and realistic form of immune intervention (30). In this study, we tested the hypothesis that a similar form of delivery of other IL-10-inducing immunoregulatory small molecule, such as curcumin, ameliorates chronic inflammation in two CID mouse models (atherosclerosis and experimental autoimmune encephalomyelitis, EAE) *via* immune system regulation.

## Materials and Methods

### Mice and Reagents

Wild-type (WT) C57BL/6 (Charles River, Portage, MI, USA) and apolipoprotein E-deficient (ApoE^-/-^) mice (B6.129P2-Apoetm1Unc/J, The Jackson Laboratory, Bar Harbor, ME, USA) were bred and housed at 22 ± 1°C under a 12 h light/dark cycle with food and water *ad libitum* and maintained under specific pathogen-free (SPF) conditions at the animal facility of the Sede de Investigación Universitaria (SIU), Universidad de Antioquia. B6.129P2-Il10tm1Cgn/J (IL-10^-/-^) mice (The Jackson Laboratory, Bar Harbor, ME, USA) were also used and kept under SPF conditions at the animal facility, Pontificia Universidad Católica de Chile. Gender- and age-matched mice were used in all experiments for all groups. At the end of experiments euthanasia was performed using ketamine/xylazine 100/10 mg/kg intraperitoneally (ip). All experiments were approved by the corresponding Institutional Animal Care and Use Committees (Comité Institucional para el Uso y Cuidado de los Animales de Experimentación, Cicual, Universidad de Antioquia; and Comité Ético Científico para el Cuidado de Animales y Ambiente, CEC-CAA, Pontificia Universidad Católica de Chile) and performed following local and international guidelines and regulations. Curcumin (≥90%) was purchased from Cayman Chemical (Item No. 81025; MI, USA), Curcumin stock solutions (20 mg/mL) were prepared in Dimethyl sulfoxide (DMSO; Sigma, D-2650) and stored at -20°C. Paraformaldehyde (PFA), sucrose, Oil Red O, hematoxylin and eosin (H&E), and ethanol were purchased from Sigma-Aldrich (St. Louis, MO, USA). PBS and RPMI were obtained from Gibco (Thermo Fisher Scientific, MA, USA).

### Assessment of the Atheroprotective Effect

8-10-weeks old male ApoE^-/-^ mice were injected sc in the hind footpad on days 0, 4 and 7, for a round of treatment with curcumin (10 µg, diluted in sterile PBS from the stock solution, final volume of 50 µl). Control mice were injected with a similar volume of vehicle (1% DMSO in PBS). This scheme was repeated eight times every other week, alternating between left and right hind footpad. Mice were shifted from a standard chow diet at day 21 (after 2 rounds of intervention) to a high-fat diet (HFD; Teklad Custom diet TD.88137, 42% of kcal from fat, 0.2% cholesterol, Envigo, Tampa, FL, USA). Body weight was monitored weekly in individual animals. One week after the last round of treatment, animals were euthanized to obtain hearts-aortas, blood, spleens, popliteal lymph nodes (LN), liver and kidneys. Spleens, livers, and kidneys were weighted and counts of total and viable splenocytes were obtained by trypan blue exclusion. Cryosections from the aortic root were used to quantify atherosclerotic lesion size and lipid deposition area by H&E and Oil red O-staining, respectively, as previously reported (30). Immunofluorescence microscopy analysis was also performed to evaluate inflammatory infiltrates of macrophages and T cells in the aortic sinus by using anti-CD68 and anti-CD3 mAb, as reported (31). Additionally, a preliminary experiment in 8-10-weeks old female ApoE^-/-^ mice fed only chow diet was also performed following a similar treatment scheme; results are presented in **Supplementary Material**.

### Serum Biochemical Analysis and Antioxidant Activity in ApoE^-/-^ Mice

Serum samples were obtained to quantify the circulating levels of total cholesterol, c-LDL, c-HDL, triglycerides, AST, ALT, ALP, LDH, glucose, creatinine, and amylases by colorimetric assay kits (Biosystems S.A., Barcelona, Spain). Quantification of the lipid peroxidation product malondialdehyde (MDA) in plasma and tissue homogenates from liver and kidney was performed by the thiobarbituric acid-reactive species method, using a 0.62 to 20 μM calibration curve of 1,1,3,3-Tetraethoxypropane as standard (32, 33). For FRAP analysis, the ability of the samples to reduce the ferric-2,4,6-tripyridyl-s-triazine complex (TPTZ-FE) was measured according to the method reported by Benzie and Szeto (34). The FRAP working solution contained 300 mM acetate buffer (pH 3.6), 40 mM TPTZ and 20 mM FeCl_3_·6H_2_O in water in a 10:1:1 ratio. Samples were obtained from tissue homogenates (liver and kidneys) or by diluting blood samples in PBS 1:1 and filtering through a 0.5 µm membrane. Samples and working solution were mixed at a ratio of 1:25 for 10 min at 37°C in the dark. The absorbance was read at 593 nm using a UV/Vis spectrophotometer (PowerWave XS2) and interpolated on a calibration curve with Trolox (31.25-1000 µM). To normalize results in tissue samples, protein quantification was performed using the bicinchoninic acid (BCA) method, which generates a detectable colorimetric reaction at 562 nm.

### Curcumin Distribution Analysis

C57BL/6 WT mice were treated sc with one injection or one round of curcumin in the hind footpad and euthanized ¼, 24, 48 or 72 h later. Whole paw and draining LN (dLN) were harvested to obtain tissue homogenates in 200 µL extraction solvent (absolute ethanol), centrifuged, and supernatants were collected. The procedure was repeated 3 times (obtaining 600 µL in total) to maximize curcumin extraction. Serum samples (~150-300 µL) were separated from coagulated total blood and mixed with 200 µL extraction solvent. The solvent was then evaporated at 55°C, dry samples were reconstituted with 100 µL of ethanol and curcumin was quantified by a fluorometric method in a BioTek Synergy HT multi-mode microplate reader (BioTek^®^ Instruments, Inc., USA) using 485 nm for excitation and 528 nm for emission. Curcumin quantity was obtained by extrapolating in a calibration curve (0.1 µg - 10 µg, expressed as curcumin mass in 100 µL). Tissues from vehicle treated mice were used as blank samples.

### Flow Cytometry and Total and ox-LDL Specific IgG and IgM Antibodies

The frequency of FOXP3^+^ CD4^+^ Treg cells was analyzed by flow cytometry on spleen and dLN cell suspensions; for intracellular cytokine staining, splenocytes were stimulated with 5 ng/mL PMA (Sigma, P-8139) and 500 ng/mL ionomycin (Sigma, I-0634) to determine the frequency of IFNγ-producing CD4^+^ cells (mostly compatible with Th1 cells), IFNγ-producing CD8^+^ cells, IL-10-producing MHCII^hi^ CD11c^+^ dendritic cells, and IL-10-producing CD19^+^ B cells as previously reported (30). TNFα-producing CD4^+^ cells (clone MP6-XT22) were also analyzed.

Total IgM and IgG were determined in serum samples by ELISA (Ready-Set-Go Kits, eBioscience, USA) following the manufacturer’s instructions. OxLDL-specific IgM and IgG were determined in serum samples by home-made ELISA assay. Briefly, 96-well plates (Costar, USA) were coated with 50 µL of ox-LDL (10 µg/mL) in PBS pH 7.4, overnight at 4°C, washed 5 times with buffer (TBS pH 8.0), blocked with 1% BSA at room temperature, incubated for 24 h with 100 µL serum samples (diluted 1:100, 1:1000 and 1:10000 in PBS-FBS 10%), and finally washed 5 times with TBS. Anti-IgM or anti-IgG mouse antibody conjugates were added (1:8000; Jackson ImmunoResearch, USA) for 2 h at 37°C. Plates were washed 5 times and the Avidin/HRP conjugate (1:250; eBioscience, USA) added for 30 min at room temperature. After washing with TBS, 100 µL of the tetramethylbenzidine substrate (BD Biosciences, USA) were added for 30 minutes and the absorbance was measured in an ELISA reader (405 nm; Bio-Rad iMark, USA) after stopping the reaction with 50 µL of a 2N H_2_SO_4_ solution.

### Assessment of the Neuroprotective Effect in the EAE Model

The effect of curcumin treatment in the EAE model was assessed on 7-10-week-old female WT and IL-10^-/-^ C57BL/6 mice. WT animals (17-20 g) were immunized sc with 0.2 mL of an emulsion containing 50 µg of myelin oligodendrocyte glycoprotein (MOG)-derived peptide (35-55; MEVGWYRSPFSRVVHLYRNGK; Syd Labs, USA) dispersed in 0.1 mL of sterile PBS and 0.1 mL emulsified incomplete Freund’s adjuvant (Sigma, USA) supplemented with heat-inactivated *Mycobacterium tuberculosis* H37 RA (1000 µg) (Sigma, USA). Emulsion was injected in both upper and hind back, 0.1 ml emulsion per injection. Two and 48 h after immunization, mice received an ip injection of 400 ng of Pertussis toxin (PTX, Sigma, USA) diluted in 200 µL PBS. IL-10^-/-^ mice received 100 µg of MOG peptide, 500 µg of heat-inactivated *Mycobacterium tuberculosis* H37 RA and 250 ng of PTX to induce a less severe EAE, since IL-10 deficiency can increase the severity of the disease (35). Mice were treated as follows: curcumin (10 µg, in a volume of 50 µL, dispersed in PBS from stock solution) or vehicle (1% DMSO diluted in PBS), administered sc into both hind footpads (10 µg/each footpad, total curcumin 20 µg/mouse) every other day. This dose and scheme of treatment were chosen in order to fit the principle of repetitive yet spaced microdose delivery with the length of the EAE model. Two different treatment schemes were used: i) a prophylactic/therapeutic scheme in which treatment started 7 days prior MOG immunization, with clinical assessment and treatment performed until day 22 or day 28; and ii) a purely therapeutic scheme in which treatment started 7 days after MOG immunization and continued until day 22.

Daily clinical scores were recorded blindly according to a standardized 0-5 scale as follows: 0, no clinical signs; 0.5, partially limp tail; 1, paralyzed tail; 2, movement coordination loss and hind limb paresis; 2.5, one hind limb paralyzed; 3, both hind limbs paralyzed; 3.5, both hind limbs paralyzed and weakness in forelimbs; 4, forelimbs paralyzed; 5, moribund (36). Mice were weighted before and every day after EAE induction and the body weight change was plotted. Daily percentage of healthy animals were graphed using a Kaplan-Meier estimator in function of the days post-immunization in which each animal became ill (score greater than 0). After 22 or 28 days of EAE induction, mice were euthanized as aforementioned and lumbar spinal cord and spleens were collected. Paraffin sections from spinal cord were obtained to assess myelin integrity and cell infiltration. Briefly, after euthanasia, mice were perfused through the heart with 30 mL 0.9% NaCl followed by 30 mL 4% PFA in PBS. The lumbar region of the spinal cord was dissected, PFA fixed, paraffin embedded, and 4 µm thick sections were stained with Luxol Fast Blue (LFB) or H&E to analyze demyelination or infiltrating cells, respectively. All sections (5 per mouse) were analyzed using a microscope (Carl Zeiss Axio Scope A1, Germany) and a Nikon DS-Fi2 (Nikon, Japan) digital video camera. The scale used to evaluate demyelination and inflammation was described elsewhere (37).

### Splenocyte Stimulation and Cytokine Assays

Splenocytes were resuspended in supplemented cell culture medium at a density of 2.0×10^6^ cells/mL in RPMI 1640 (GlutaMAX), containing 10% FBS, 100 U/mL penicillin, 100 µg/mL streptomycin (Gibco, Thermo Fisher Scientific, MA, USA) and stimulated with MOG peptide (20 µg/mL) during 72 h at 37°C, 5% CO_2_. Supernatants were collected and the concentration of IFNγ, TNFα (Mouse ELISA Kit II OptEIA, BD Biosciences, USA), IL-17, and IL-10 (R&D Systems, USA) was analyzed by ELISA.

### Statistical Analysis

Data were tabulated, plotted, and analyzed using GraphPad Prism 8.3 (GraphPad Software Inc., San Diego, CA, USA). In some graphs, data is presented using a “box and whiskers” plot in which box hinges ranged from the 25^th^ to the 75^th^ percentile and the median (line), mean (+) and min/max data (whiskers) are represented. For the comparison of two unpaired groups, the Mann-Whitney *U* test was used. To take account of multiplicity in group comparisons, two-way ANOVA followed by Bonferroni’s post-test was conducted. The percentage of healthy animals was plotted as a Kaplan-Meier curve and analyzed with a Mantel-Cox test. To assess the relationship among multiple variables in the EAE experiments, a Spearman rank correlation analysis was performed. Results are presented in figures as the mean or the mean ± SEM. P<0.05 was considered significant (*P<0.05; **P<0.01; ***P<0.001; ****P<0.0001).

## Results

### Subcutaneous Repetitive Microdose Delivery (REMID) of the Polyphenol Curcumin Exerts Atheroprotective Effects Related to Immune Modulation but Not to Metabolic or Antioxidant Activities

**Figure 1:**
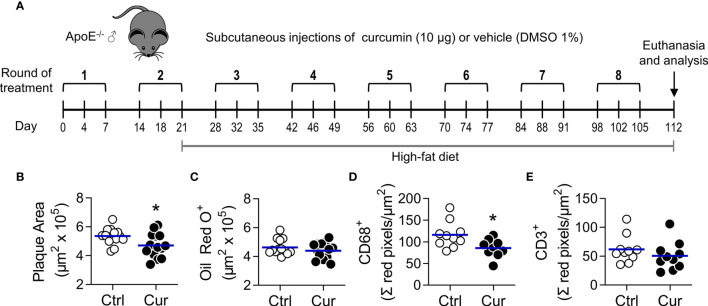
Subcutaneous microdose delivery of curcumin attenuates atherosclerosis development in dyslipidemic ApoE^-/-^ mice. **(A)** ApoE-/- mice were treated three times a week (1 round of treatment), with subcutaneous injections of curcumin (Cur, 10 µg) or vehicle (Ctrl, DMSO 1%) for 8 rounds every other week, and fed a high-fat diet (HFD), as shown. Animal euthanasia was performed on day 112, and samples were collected and processed as indicated in Materials and Methods. **(B, C)** Cryosections from the aortic root were stained with **(B)** H&E and **(C)** Oil red O, to quantify plaque area and lipid deposition, respectively. **(D, E)** The extent of **(D)** macrophage (CD68^+^) and **(E)** T cell (CD3^+^) infiltration in aortic root cryosections was also assessed by immunofluorescent staining. The areas of the atheromatous lesions, lipid deposition, as well as the macrophage and T cell infiltrates were calculated with an image analysis software (see *Materials and Methods*). Each point represents the average area per mouse (7-9 sections/mouse), and bars represent the mean of 10-15 mice. *P<0.05 (Mann-Whitney *U* test). In repetition experiments, similar reductions in the atheromatous plaque area were observed (**Supplementary Figure S1**).

In a previous proof of concept report, we proposed a novel immunomodulatory strategy consisting in the local low-dose delivery of an IL-10-inducing formulation (containing VitD/Dexa) as a form of immune intervention for atherosclerosis (30). Since other hydrophobic small molecules have similar tolDC/Treg-inducing properties (23–26, 38–40), we postulated that after a similar dose and form of administration of any of them, an atheroprotective effect should be reproduced. To test this hypothesis, atheroprotection was evaluated in male ApoE^-/-^ mice treated with microdoses of the polyphenol curcumin (10 µg/mice, approximately 0.4 mg/kg) delivered sc, three times a week, every other week, for 8 rounds of treatment (**Figure 1A**). Results indicated that compared to control animals, mice receiving curcumin injections exhibited significantly reduced atheromatous plaque size (**Figure 1B** and **Supplementary Figure S1**), although lipid content remained unchanged as assessed by Oil Red O staining (**Figure 1C**). CD68 and CD3 immunostaining of serial sections from the aortic root indicated that curcumin-induced atheroprotection was accompanied by a significant decrease of macrophage but not T cell infiltration in the atherosclerotic lesions (**Figures 1D, E**). These results confirmed that the atheroprotective effect of our novel sc REMID strategy is not restricted to the VitD/Dexa formulation, but also observed when using other IL-10/Treg-inducing hydrophobic small molecule such as curcumin.

**Figure 2:**
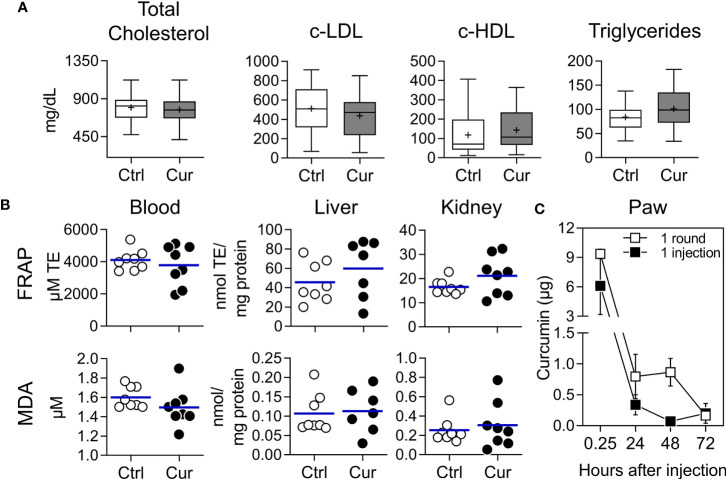
Absence of systemic lipid-lowering or antioxidant effects in ApoE^-/-^ mice treated with subcutaneous microdose curcumin. **(A, B)** Dyslipidemic ApoE^-/-^ mice were treated with curcumin (Cur) or vehicle (Ctrl) as shown in **Figure 1A**. **(A)** Total cholesterol, c-LDL, c-HDL, and triglycerides were measured in serum samples at the end of the experiment. **(B)** The antioxidant effect of curcumin *in vivo* was assessed by preparing blood, liver and kidney homogenates and evaluating the antioxidant capacity with the FRAP method (upper panel) or quantifying the concentration of the lipid peroxidation products MDA (lower panel) for each matrix. Results presented in **(A)** correspond to two pooled experiments (n=27 mice/group) and plotted as box and whiskers. Results in **(B)** correspond to one experiment (n=7-8 mice/group) and each dot represents an individual animal and the line the mean for each group. **(A, B)** Mann-Whitney *U* test was performed resulting in the two treatment groups not being statistically different at any measurement. **(C)** For the biodistribution experiment, groups of WT C57BL/6 mice were injected sc with curcumin (10 µg) in the hind footpad, euthanized ¼, 24, 48 and 72 h after one injection or one round of treatment (see **Figure 1A**), and the amount of curcumin recovered from the whole paw, dLN and serum homogenates was determined by fluorescence spectrometry (see *Materials and Methods* for details). Mice injected with vehicle were used as blanks. Results are presented as mean ± SEM for each timepoint (n=3). Curcumin amounts in dLN and serum samples were below the limit of quantification of the technique (0.1 µg; data not shown).

Several studies have already reported curcumin to be atheroprotective in animal models (41–43). Given the poor pharmacokinetics of the polyphenol (44, 45), a high dose (3-1500 mg/kg, oral/ip/iv) is often selected in order to maximize the opportunity to achieve significant atheroprotection, which has been related to systemic lipid-lowering, antioxidant, and anti-inflammatory activities. In contrast, our strategy is based on a very low dose (0.4 mg/kg) sc administration, intended to restrict the actions of the agent to the site of injection and dLN, rather than to attain direct systemic effects (30). Our results indicate that curcumin intervention did not alter the blood levels of total cholesterol, c-LDL, c-HDL, and triglycerides (**Figure 2A**). Additionally, curcumin treatment did not enhance the antioxidant activity, as measured by FRAP (**Figure 2B**, upper panel), or the amounts of the peroxidation product MDA (**Figure 2B**, lower panel) in blood or tissue homogenates from liver and kidney. In line with these findings, a preliminary analysis of curcumin biodistribution upon sc delivery in WT mice showed that after 15 min most of the administered compound was recovered from the injection site, with curcumin levels remaining within the test detection range up to 24 h when only one injection was given. After completion of one round of treatment, the compound was locally detected up to 48 h at amounts of approximately 1 µg/paw (**Figure 2C**). In contrast, and as anticipated, curcumin levels in serum samples were below the detection threshold of the fluorescent method. Unexpectedly, curcumin was not detected in dLN. Consistent with these results, no evidence of clinical, macropathological, biochemical, and immune toxicity was observed in curcumin-treated ApoE^-/-^ mice (**Supplementary Figure S2**). Thus, curcumin-mediated atheroprotection does not appear to be a consequence of the systemic distribution of the polyphenol and its subsequent direct effects on lipid metabolism or antioxidant activity in internal organs.

**Figure 3:**
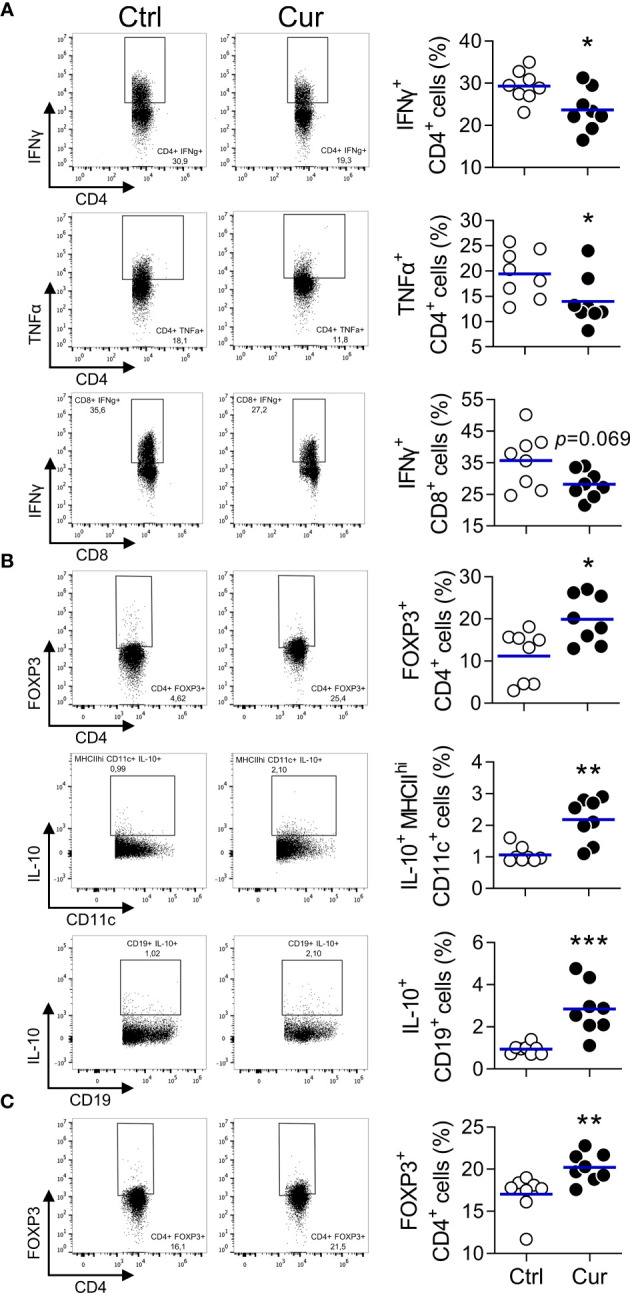
Curcumin-induced atheroprotection associates with attenuated Th1 and increased regulatory response. **(A, B)** Spleen cell suspensions from curcumin- or vehicle-treated mice from **Figure 1** were stained with appropriate mAbs to determine the frequency of **(A)** IFNγ^+^ CD4^+^, TNFα^+^ CD4^+^, and IFNγ^+^ CD8^+^ cells, as well as **(B)** FOXP3^+^ CD4^+^, IL-10^+^ MHCII^hi^ CD11c^+^ DC and IL-10^+^ CD19^+^ B cells by flow cytometry. **(C)** Popliteal lymph node (pLN) cell suspensions were also obtained and the frequency of the FOXP3^^+^^ CD4^^+^^ cell population quantified. Left panel shows representative dotplots. Each dot in the right panel represents an individual animal, and the line represents the mean from 8 mice/group. *P < 0.05; **P < 0.01; ***P < 0.001 (Mann-Whitney *U* test).

Next, we explored whether REMID curcumin had immunomodulatory effects. On one hand, we observed that the frequencies of splenic CD4^+^ cells producing the Th1 proinflammatory/proatherogenic cytokines IFNγ and TNFα were significantly reduced in curcumin-treated mice compared to vehicle-treated animals, and a similar trend was also evidenced for IFNγ-producing CD8^+^ cells (**Figure 3A**). In contrast, an enhanced immunoregulatory/atheroprotective response was observed in curcumin-treated mice, since the frequency of FOXP3^+^ CD4^+^ Treg cells, IL-10-producing DC (compatible with tolDC), and IL-10-producing B cells (compatible with Bregs) were significantly higher (**Figure 3B**). The assessment of IL-10-producing CD4^+^ cells, Tr1-like, was inconclusive, since independent experiments shed increased, reduced or no significant differences in its frequency (not shown). These results demonstrate that sc curcumin delivery shifted the splenic immune response towards a less proatherogenic and more atheroprotective balance. Moreover, despite the limited number of cells recovered from the popliteal lymph node (pLN), we could demonstrate that the treatment also promoted FOXP3^+^ CD4^+^ Treg cells in this lymphoid organ draining the site of curcumin delivery (**Figure 3C**). Recent reports indicate that IgM secreted by B cells play an important role in atheroprotection (46); however, in the present work, we did not observe differences in total or oxLDL-specific IgM or IgG antibodies (**Supplementary Figure S3**). Altogether, results presented thus far indicate that sc REMID curcumin does not induce systemic lipid-modifying or antioxidant effects which is consistent with its limited blood circulation and distribution; however, despite the local low dose delivery, an immunoregulatory activity was observed that could be related to its atheroprotective effect. All these observations are strikingly similar to those described with VitD/Dexa (30), and validate our approach with a safer, simpler, and accessible tolDC/Tregs-inducing hydrophobic small molecule formulation.

### Subcutaneous REMID Curcumin Also Triggers Immunoregulation and Promotes Neuroprotection in an EAE Model

**Figure 4:**
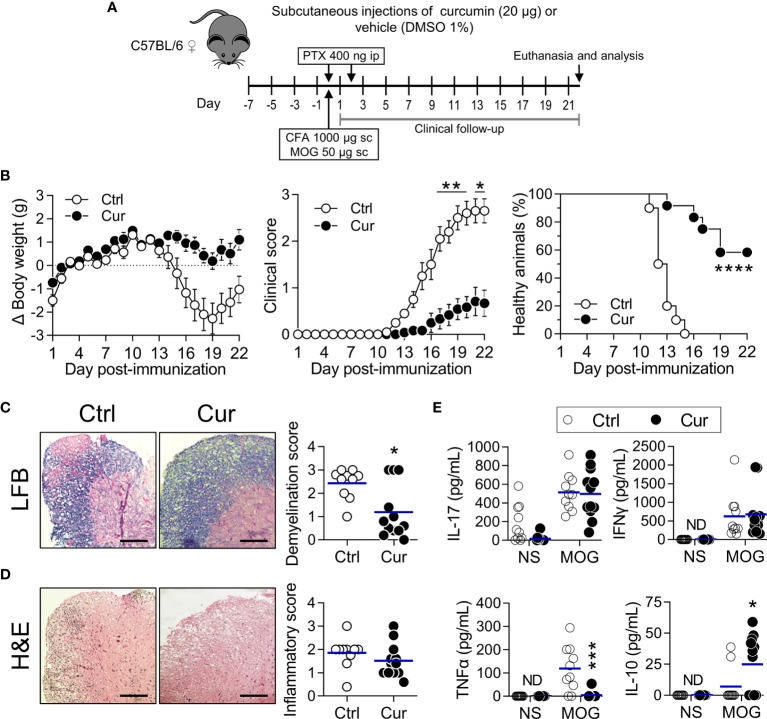
Subcutaneous microdose curcumin is also neuroprotective. **(A)** WT C57BL/6 female mice were treated subcutaneously in the hind footpads every other day with curcumin (Cur, 10 µg each footpad, 20 µg per mouse, approximately 0.8 mg/kg) or vehicle (DMSO 1%; Ctrl), 7 days prior EAE induction (day 0) and until the end of the experiment, as shown. Clinical follow-up was performed daily, and after euthanasia (day 22), samples were collected to perform histopathological analysis and immunological tests. **(B)** Changes in body weight (left), clinical score (center) and percentage of healthy animals (right) were recorded daily after immunization (see *Materials and Methods* for details). Data is presented as the mean ± SEM (left and center). Percentage of healthy animals (right) was plotted as a Kaplan-Meier curve. **(C, D)** Representative images of the lumbar cord sections stained with **(C)** LFB and **(D)** H&E. Plotted data represents the **(C)** demyelination score and **(D)** inflammatory score obtained after image analysis from each animal in both treatment groups. Scale bar=25 µm. Each point represents individual animals and bars represent the mean of the group. **(E)** 2.0×10^6^/mL splenocytes were stimulated with MOG (20 µg/mL) or PBS (NS) for 72 h. The amounts of the indicated cytokine produced by cells in response to stimuli were quantified in supernatants by ELISA. ND, not detected. Results are plotted as individual animals and mean (n=10-12). *P < 0.05; **P < 0.01; ***P < 0.001; ****P < 0.0001. Statistical tests were: (**B**, left and center) Two-way ANOVA for repeated measures with Bonferroni’s post-test, (**B**, right) Mantel-Cox test and **(C–E)** Mann-Whitney *U* test.

Having demonstrated that sc microdose curcumin is atheroprotective, we next investigated whether other inflammatory diseases, for which the stimulation of tolDC/Tregs has therapeutic potential, are favorably impacted by our REMID strategy. We chose the EAE model given the immunopathological convergences of atherosclerosis with autoimmune diseases (47–49), its preclinical value as a tool for assessing therapeutic interventions (50, 51), and the possibility to address practical and mechanistic questions in a substantially shorter amount of time. We found that microdose curcumin treatment (**Figure 4A**) exerted clinical neuroprotection evidenced by attenuated weight loss and improved clinical score (**Figure 4B**, left and center). We also noticed that a higher percentage of animals (58%) did not develop the clinical manifestations of the disease when treated with curcumin (clinical score=0), while all vehicle-treated mice became ill within 15 days post-immunization (**Figure 4B**, right). In a repetition experiment with an extended treatment scheme and clinical follow-up (**Supplementary Figure S4A**), curcumin exhibited a similar neuroprotective effect (**Supplementary Figure S4B**).

Histopathological analysis of the spinal cord revealed a clear attenuation of demyelination, an important sign of active disease (**Figure 4C** and **Supplementary Figure S4C**). The inflammatory score was also lower in curcumin-treated mice, although it required an extended treatment to evidence a statistically significant reduction (**Figure 4D** and **Supplementary Figure S4D**). Interestingly, the treatment significantly promoted IL-10 and abrogated TNFα production upon MOG stimulation of splenic cells (**Figure 4E**), confirming the immunoregulatory and IL-10-inducing capacity of our strategy in a neuroinflammation model. The repetition experiment showed a non-identical but similar immunoregulatory effect of curcumin, with a significantly lower secretion of IL-17 and a mild tendency to produce less IFNγ and more IL-10 in treated animals (**Supplementary Figure S4E**). A Spearman correlation analysis allowed us to verify the relationship between these parameters, indicating that IL-10 induction moderately, but significantly, correlated with disease amelioration (**Supplementary Figure S5**). By putting together these observations, it is evident that our sc REMID curcumin also promotes clinical and histopathological neuroprotection apparently through mechanisms that involve immune regulation.

### The Neuroprotective Effects of Curcumin Require IL-10 and Can Be Promoted After Disease Induction

**Figure 5:**
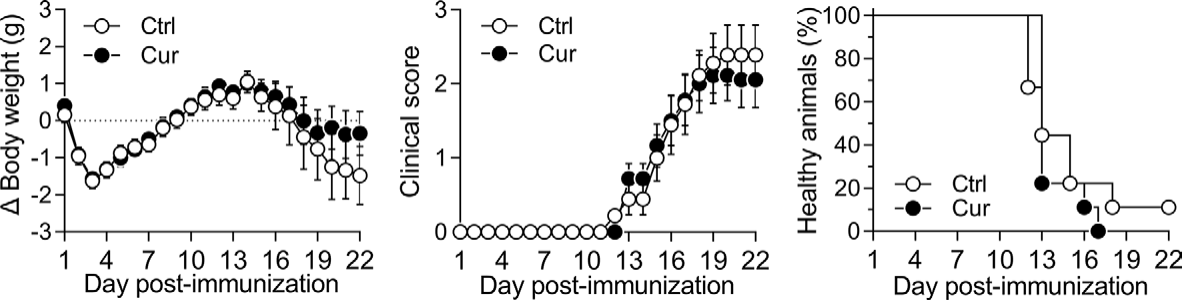
Neuroprotective effect of curcumin is IL-10 dependent. IL-10^-/-^ mice were treated with vehicle (Ctrl) or curcumin (Cur) as shown in **Figure 4A** with minor modifications (see *Materials and Methods* for detailed explanation). After immunization, animals were monitored daily and changes in body weight (left), clinical score (center) and percentage of healthy animals (right) were recorded. Data is presented as the mean ± SEM and a two-way ANOVA for repeated measures with Bonferroni’s post-test was performed (left and center). Percentage of healthy animals was plotted as a Kaplan-Meier curve and a Mantel-Cox test was performed (right). n=9 animals per group. No statistically significant differences were found between Ctrl and Cur groups.

Considering that proinflammatory/anti-inflammatory mediators balance plays a critical role in regulating inflammatory and autoimmune diseases, and that our curcumin delivery strategy promotes IL-10 in both experimental models, atherosclerosis (**Figure 3B**) and EAE (**Figure 4E**), we took advantage of the latter in the sense that a single knock-out mouse strain would suffice to test the IL-10 requirement. Thus, EAE was induced in IL-10^-/-^ mice as described in the Materials and Methods section. Following the treatment scheme depicted in **Figure 4A**, we found that in the absence of IL-10 neuroprotection was abrogated (**Figure 5**), further confirming that induction of IL-10 by sc REMID curcumin is pivotal to attain this beneficial effect.

**Figure 6:**
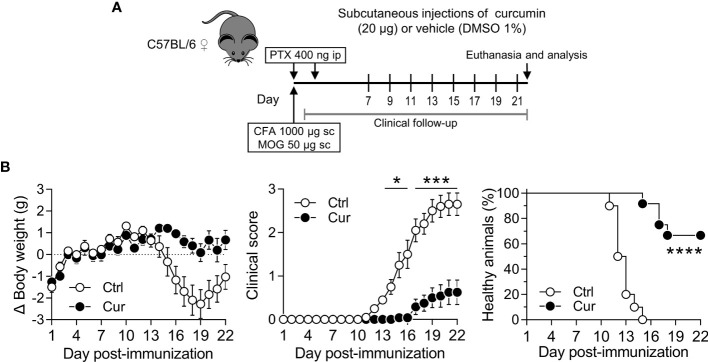
Microdose curcumin is also neuroprotective on a therapeutic scheme of administration. **(A)** WT C57BL/6 female mice were MOG-immunized and treated ip with PTX to induce EAE. From day 7 until the end of the experiment (day 22), mice received sc curcumin (Cur) or vehicle (Ctrl) every other day. Clinical follow-up was performed daily during the length of the experiment. **(B)** Changes in body weight (left), clinical score (center) and percentage of healthy animals (right) were recorded daily after immunization. Data is presented as the mean ± SEM and a two-way ANOVA for repeated measures with Bonferroni’s post-test was carried out (**B**, left and center). Percentage of healthy animals was plotted as a Kaplan-Meier curve and a Mantel-Cox test was performed (**B**, right). n=11 animals per group. *P < 0.05; ***P < 0.001; ****P < 0.0001.

From a translational perspective, it is enormously valuable to test if an immune intervention is effective when the disease is already progressing in the preclinical model. In this regard, we wanted to explore whether the use of curcumin on a purely therapeutic scheme (**Figure 6A**) was also neuroprotective. Interestingly, we found that only 8 sc injections of microdose curcumin, starting at day 7 post-immunization, promoted amelioration of clinical signs such as weight loss and clinical score, as well as a higher percentage of healthy animals that did not developed the disease (**Figure 6B**). Overall, this set of experiments demonstrates that the immune intervention strategy described herein, using the polyphenol curcumin as an IL-10-inducing-small-molecule model and EAE as a disease model, is effective at controlling inflammatory symptoms through an IL-10-dependent mechanism. This strategy was also effectual after disease triggering, highlighting its translational value.

## Discussion

In the face of current demographic/environmental changes and unhealthy lifestyle behaviors that trigger dysregulated immune responses and chronic inflammation, restoring the immune balance, by prompting the return to homeostasis, has become central in attaining organismal well-being. Thus, addressing the dysregulated immune function rather than blocking inflammatory mediators offers the opportunity to design new interventions intended to reinforce endogenous regulatory mechanisms that are undermined when inflammation is engaged and sustained as a chronic process (52). Based on a recent study from our group, in which we showed that low dose locally delivered VitD/Dexa was effective at halting the progress of atherosclerosis, *via* induction of IL-10-producing regulatory leukocytes instead of prompting direct systemic effects (30), here we hypothesized that other immunoregulatory small molecules administered on a similar sc REMID strategy should also be able to restore the pro/anti-inflammatory balance and impact disease. From numerous synthetic and natural regulatory leukocyte-inducing small molecules (23–26, 38–40), we chose curcumin as a model molecule based on its immunomodulatory capacities [IL-10- and tolDC/Treg-induction *in vitro* and *in vivo* (26, 53, 54)], its physicochemical characteristics [very low water solubility and tendency to aggregate (55)], and its wide availability at an extremely low cost (56). In the present report, we extended our novel strategy by confirming that i) a curcumin-based formulation is also atheroprotective when following the same dose, route, and scheme of administration, ii) the formulation was also protective in a second relevant animal model of CID, such as autoimmune neuroinflammation, and iii) the neuroprotective effect depends on the production of IL-10 and could also be elicited after disease triggering.

As with VitD/Dexa (30), we found that sc REMID delivery of curcumin was atheroprotective, as evidenced by the size of the atheromatous lesions and macrophage infiltrate in the aortic root (**Figure 1**). The fact that curcumin is atheroprotective is not a novel finding, since several publications have already reported this effect mostly using dietary/oral high doses (41). What significantly differs between this study and those reports is the rationale and approach. While other studies intended to exploit curcumin in the classical pharmacological manner, in which high doses are used to try to overcome its poor oral bioavailability, our strategy took advantage of the limited solubility of curcumin to direct its actions to the immune cells in the interstitial space at the site of injection and dLN. Thus, we intentionally seek to limit systemic distribution by using the sc route and a very low dose. The dose of curcumin used in oral/dietary studies ranged from ~10 to ~1500 mg/kg/dose (a total amount of ~14-4200 mg per mouse in the experiment) (41), which expectedly, was largely excreted in the feces with only a minimal amount reaching circulation to promote putative protective systemic effects (45, 57, 58). This contrasts with our microdose treatment (0.4 mg/kg/dose; total amount of 0.24 mg per mouse in the experiment), which was sufficient to significantly exert protection (**Figure 1** and **Supplementary Figure S1**). Results from a pilot experiment using female ApoE^-/-^ mice fed a chow diet that received 4 or 7 rounds of treatment **(** **Supplementary Figure S6**) showed a trend but not a significant reduction of plaque area, suggesting that extended exposure to sc microdose curcumin, for at least 8 rounds, is a requirement for significant atheroprotection. Therefore, these results confirmed that just by shifting from the oral to the sc route, in order to deliver the compound locally rather than systemically to internal organs/targets, reproducible atheroprotection can be achieved with a significantly lower amount of the compound (10^2^ to10^4^-fold less curcumin).

Assessment of serum lipids profile showed no differences between curcumin- and vehicle-treated mice. This was observed not only in HFD-fed male ApoE^-/-^ mice (**Figure 2A**), but also in female ApoE^-/-^ mice fed a chow diet **(** **Supplementary Figure S6**). Although redox imbalance associates with the onset and progression of atherosclerosis (59), and it has been shown that curcumin reduces the oxidative stress index in plasma, liver, and kidneys (60, 61), when we tested common indicators of the systemic redox status no differences were found (**Figure 2B**). Clearly, our results contrast with most studies involving dietary/oral curcumin that generally report direct lipid-lowering and antioxidant effects as the underlying mechanism of atheroprotection (41). Although the amounts of curcumin and its metabolites circulating systemically have been repeatedly reported as negligible, owing to their poor pharmacokinetics (42, 45), the improved lipid profile and antioxidant activities have been interpreted as a confirmation that the molecule was distributed systemically to key internal organs at biologically relevant concentrations. Here we show that the low dose sc curcumin-induced atheroprotection cannot be explained by the metabolic and antioxidant effects typically observed in previous reports (41), confirming that, as with VitD/Dexa (30), a distinct mechanism of action was engaged. Overall, our results reinforce the concept that curcumin direct systemic effects are not a prerequisite for atheroprotection and that, by following the sc REMID strategy instead of the oral route, the requirement of very high dose and/or pharmacokinetic improvement is bypassed.

In an attempt to directly confirm our hypothesis that sc low dose administration preferentially concentrates curcumin at the site of injection rather than favoring systemic exposure, we took advantage of its intrinsic fluorescent properties to perform a preliminary quantitation. Our findings suggest that several rounds of sc curcumin administration would ensure the exposure of resident immune cells to immunologically relevant amounts of curcumin at the treatment site (footpad). We speculate that after every injection, cells are subjected to a burst-like release of curcumin at least for 24 h, a phenomenon occurring three times per round. Interestingly, 48 h after completing one round of treatment, about 1 μg of the compound remained in the footpad, extending the local exposure beyond the round (**Figure 2C**). Since curcumin dispersion in DMSO-PBS results in the formation of large aggregates (**Supplementary Figure S7**), we presume that deposits might be occurring at the interstitial space, possibly contributing to extend its biological effects by passively targeting immune cells locally or at the draining site, a hypothesis that needs to be addressed in future experiments. However, and unexpectedly, curcumin could not be detected in the dLN, probably due to a technical limitation related to its small size for harboring an amount of curcumin sufficient to be detected by the technique used. It is also possible that curcumin had reached the dLN but it was rapidly “consumed” by resident/migratory cells, or further drained through the lymphatic system without being detected at the timepoints analyzed. On the other hand, expectedly, curcumin could not be detected in serum, which might explain the absence of direct systemic antioxidant and lipid-lowering effects. Many reports have confirmed that circulating curcumin and the amount —and proportion of the initial dose— that reaches various tissues after ip administration of this molecule is extremely low (45, 58, 62, 63). Since the quantification limit of our method was relatively high (0.1 µg), a more robust and sensitive analysis aimed to determine not only curcumin but also its metabolites and degradation products is required. Nevertheless, considering curcumin’s poor pharmacokinetics (i.e. low water solubility and stability, rapid metabolism/excretion, short half-life) (42, 45), and the very low doses used by our strategy, we do not anticipate curcumin reaching immune cells at key internal organs such spleen or target inflamed tissues (45, 62, 64).

The chronic inflammatory response is considered an important player in atherosclerosis and multiple sclerosis (MS)/EAE pathophysiology. Current evidence indicates that as inflammation intensifies, the atherosclerotic process and neuropathology aggravates (65–67). Consistently with our working hypothesis (30), we observed that microdose sc curcumin treatment promoted a more balanced proinflammatory/regulatory response, in both atherosclerosis and EAE models, as indicated by a lower frequency of Th1 cells (**Figure 3A**), mitigation of proinflammatory Th1/Th17 responses (**Figure 4E** and **Supplementary Figure S4E**), increased frequency of IL-10-producing tolDC/Bregs and Tregs (**Figures 3B, C**), upregulated IL-10 production (**Figure 4E**), and significant atheroprotection (**Figure 1**) and neuroprotection (**Figures 4B–D** and **Supplementary Figure S4B–D**). These results are in agreement with abundant literature demonstrating the atheroprotective/neuroprotective effect of IL-10 and regulatory leukocytes (Tregs, tolDC, Bregs) (11–13, 46, 67–71). Notwithstanding the fact that IL-10 induction has been proposed as a pillar of curcumin immunomodulatory actions (26, 53), surprisingly IL-10 induction has not been consistently addressed in preclinical studies exploring the potential of curcumin for atherosclerosis or MS/EAE treatment. Here we show that our novel approach did harness IL-10 curcumin inducing capability, and that protection was dependent on IL-10 induction since neuroprotection was completely abrogated in IL-10 deficient mice (**Figure 5**). This finding agrees with a previous work showing that curcumin treatment contributes to reestablishing pro/anti-inflammatory balance in lymphoid organs and CNS through the induction of IL-10-producing CD4^+^ T cells (72). In atherosclerosis, even though curcumin-induced IL-10 has not been explored previously, atheroprotective effects of high dose oral curcumin were related to Th2/Treg balance modulation in asthmatic ApoE^-/-^ mice (73). Moreover, other published preclinical studies assessing the effects of curcumin treatment in atherosclerosis/EAE models have reported a decrease of proinflammatory mediators, a phenomenon ascribed to the direct targeting of metabolic/cellular/molecular pathways, systemically or in the affected tissues (41, 74, 75). Nevertheless, yet again, all these beneficial effects were only engaged after a daily oral or ip administration of high dose curcumin (41, 76–78), a strategy which significantly contrasts with our microdose and repetitive, yet spaced, local delivery approach that limits curcumin systemic distribution. Collectively, this highlights the significance of our results since we were able to induce protection in two different and relevant CID models.

Signaling through the IL-10/IL-10R axis is of pivotal importance for the maintenance of immunological tolerance/homeostasis and for restoring the effector/regulatory balance during immune responses (18). Many innate and adaptive leukocytes can express not only IL-10 but also IL-10R, forming a network in which tolerogenic signals are transmitted, maintained, and reinforced through autocrine/paracrine feedback loops (22). Although this cytokine is considered an unparalleled candidate for targeted immunomodulation in CID (21, 22, 79), its context-dependent and paradoxical immunostimulatory effects, as well as the pharmacological problems associated with systemic rIL-10 administration appear to be accounted for the poor safety/efficacy outcomes observed in clinical trials (18, 21, 22), further indicating that novel strategies are required to harness IL-10 therapeutic potential. Even though more sophisticated approaches have been proposed (16, 80), these are difficult to envision in the widespread clinical practice, owing to their technical complexities and/or inherent safety problems (16). A more convenient strategy with the potential to generate accessible medicines for the large and growing number of patients with CID is the identification of small molecules that promote endogenous IL-10 production. Many reported small molecules with proinflammatory cytokine inhibitory activity, such as glucocorticoids, rapamycin or VitD, among others, are also endowed with the ability to promote IL-10 production *in vitro* and/or *in vivo*, and to alleviate CID in preclinical models (24, 28, 29). Those prototypical as well as more recently identified IL-10-inducing small molecules (81), however, are usually conceived on the basis of the classical drug development approach that requires particular physicochemical features such as water solubility and stability, to ensure wide distribution and sufficient systemic exposure to the therapeutic agent. This allows beneficial direct effects on the inflamed tissue, but also can be related to non-uncommon safety issues such as off-target events and organ toxicity. Our view bypasses these classical requirements since shifting to sc delivery favors the rapid and preferential exposure of cells to the agent in the interstitial space and in dLN, subsequently promoting indirect systemic effects. The results presented here not only propose new forms to exploit curcumin’s potential for the treatment of atherosclerosis or MS/EAE, but also support a novel strategy that could enable many available small molecules as possible alternatives for the immune intervention of CID, in spite of being disesteemed as potential pharmaceuticals on the basis of their “poor drugability” (64). Whether other IL-10-inducing small molecules can recapitulate these effects *in vivo* when using the REMID approach, and the spectrum of CID that can be impacted, is to be investigated in future projects.

The *in vivo* promotion of IL-10-producing cells in both models (**Figures 3** and **4**), and more strikingly, that IL-10 expression was a key requirement for neuroprotection (**Figure 5**), support a causal relationship between the IL-10 induction and the efficacy of the treatment. Based on the tolerogenic network typically promoted by IL-10 in immune cells, it is tempting to speculate that curcumin promotes sequential IL-10 expression events in different leukocyte subsets, in which the primary response of a type of leukocyte is followed by secondary IL-10 triggering in other subsets. As with VitD/Dexa (30), we hypothesize that the very low water solubility of curcumin could provoke the formation of aggregates with a wide range of particle size (**Supplementary Figure S7**) that can preferentially be captured by phagocytes present at the injection site and dLN, promoting the early conditioning of DC, macrophages, or monocytes to produce IL-10. Curcumin-triggered IL-10 production in these APC could be sufficient to “transmit” a tolerogenic signal to T cells and other leukocytes regionally and systemically. Therefore, it is possible that a tolerogenic boost at the local level would revert to a more regulatory/homeostatic environment in other lymphoid organs and inflamed tissues, contributing to regain the organismal balance (19). The presence of regulatory leukocytes both in spleen and dLN (**Figures 3B, C**), as well as the reduction of the inflammatory infiltrate at the lesion site (**Figures 1D**, **4D** and **Supplementary Figure S4D**), is consistent with this working model. How regionally formed regulatory leukocytes transmit tolerogenic signals to internal organs, including inflamed tissues where their actions are required, is intriguing, and although technical limitations prevented us from quantifying circulating regulatory leukocytes in our experiments, we speculate that vascular trafficking from the site of induction to internal organs could be the event bridging those spatially distant events. Future work will be necessary not only to elucidate the key cell sources of IL-10 and targets of IL-10R signaling, but also the spatiotemporal dynamics of the induced regulatory leukocytes, allowing to delineate the precise immunological mechanism of athero/neuroprotection described here after the treatment with microdose curcumin.

Another interesting possibility that deserves further experimental exploration is that the cells targeted directly by curcumin are not the primary source of IL-10, but rather the intermediates that trigger IL-10 production. Taking into consideration that a prominent effect of curcumin on mammalian cells is the induction of cell stress and death (82), it was not surprising the finding of more apoptotic cells in the dLN but not in the spleen of curcumin-treated compared to vehicle-treated mice in our preliminary experiments (data not shown). This observation is of interest for two reasons: first, because it reinforces the idea of negligible systemic distribution of curcumin and preferential local effects. And second, because curcumin aggregates in the biophase at the injection site might ensure sustained local induction of apoptotic cells, and their subsequent non-inflammatory removal *via* efferocytosis, a process known to trigger IL-10 production and tolerance induction (83, 84). Moreover, macrophages and DC are more efferocytic when exposed to curcumin (85, 86), implying that curcumin is endowed not only with the capacity to generate efferocytic ligands but also to promote the efferocyte activity. Thus, we postulate that both local phagocyte conditioning *via* direct curcumin actions and the indirect conditioning *via* apoptotic cell induction and removal, might act in concert to boost tolerogenic effects such as IL-10 production in our experimental setting.

Collectively, the results presented here, together with our previous study (30), propose a novel and more rational approach to promote the restoration of the pro/anti-inflammatory balance, by exploiting the immunoregulatory properties of curcumin and other IL-10-inducing small molecules and bypassing the high dose and systemic exposure requirements. In our strategy, endogenous IL-10 is induced *via* microdose local delivery of hydrophobic small molecules such as curcumin, VitD or Dexa, avoiding the inconveniences of systemic drugs or exogenous IL-10 supplementation. Considering that low-cost treatments for globally impacting the CID burden are urgently needed (87), here we present an approach with a high translational perspective that uses inexpensive, well known, and widely available molecules that could be reformulated for managing CID prophylactically and/or therapeutically.

## Data Availability Statement

The raw data supporting the conclusions of this article will be made available by the authors, without undue reservation.

## Ethics Statement

The animal study was reviewed and approved by Comité Institucional para el Uso y Cuidado de los Animales de Experimentación, Cicual, Universidad de Antioquia; and Comité Ético Científico para el Cuidado de Animales y Ambiente, CEC-CAA, Pontificia Universidad Católica de Chile.

## Author Contributions 

JR-P conceived, designed, and directed the study. JT-G, JJ, LO-Q, CP-O, NG-V, DB-E, EC-G, and JR-P designed and planned the experiments. JT-G, JJ, and LO-Q conducted the experiments. JT-G, JJ, NG-V, DB-E, EC-G, and JR-P analyzed the results and interpreted the data. CC, AR-D, LD, PG, SB, CR, and AK provided material, tools, and/or expertise for the experiment with IL-10-deficient mice. DB-E, EC-G, and JR-P wrote the manuscript. JT-G and JJ contributed to manuscript preparation. All authors contributed to the article and approved the submitted version.

## Funding

This work was supported by Colciencias/Ministerio de Ciencia, Tecnología e Innovación de Colombia (Grants 1115-807-63108, 1115-519-28906, and 1115-343-19225) and Universidad de Antioquia. Additional support was received from Estrategia de Sostenibilidad-CODI (Universidad de Antioquia); Fondo de Ciencia, Tecnología e Innovación del Sistema General de Regalías de Colombia No. 137C-2014; the Millennium Institute on Immunology Immunotherapy (ICN09_16; P09/016-F); the ANID PAI project I781902009 and the FONDECYT 1190864 from ANID-Chile; Fondo Nacional de Desarrollo Científico y Tecnológico (Fondecyt) #1170964 and #1190830. JT-G is recipient of a doctoral scholarship from Colciencias/Ministerio de Ciencia, Tecnología e Innovación de Colombia (Convocatoria 617-2014).

## Conflict of Interest

The authors declare that the research was conducted in the absence of any commercial or financial relationships that could be construed as a potential conflict of interest.

## Abbreviations

ALP, Alkaline Phosphatase; ALT, Alanine Transaminase; APC, Antigen-Presenting Cell(s); ApoE^-/-^, Apolipoprotein E-deficient; AST, Aspartate Transaminase; Bregs, Regulatory B Cells; CD, Cluster of Differentiation; CFA, Complete Freund’s Adjuvant; CID, Chronic Inflammatory Diseases; CNS, Central Nervous System; Ctrl, control (vehicle); Cur, Curcumin; CVD, Cardiovascular Diseases; DC, Dendritic Cell(s); Dexa, Dexamethasone; dLN, draining Lymph Node; DMSO, Dimethyl Sulfoxide; EAE, Experimental Autoimmune Encephalomyelitis; ELISA, Enzyme-Linked Immunosorbent Assay; FOXP3, Forkhead Box P3; FRAP, Ferric Reducing Antioxidant Power; HDL, High-Density Lipoproteins; HFD, High-Fat Diet; H&E, Hematoxylin & Eosin; IFNγ, Interferon gamma; Ig, Immunoglobulin; IL, Interleukin; IL-10R, IL-10 Receptor; ip, intraperitoneal; iv, intravenous; LDH, Lactate Dehydrogenase; LDL, Low-Density Lipoproteins; LFB, Luxol Fast Blue; LN, Lymph Node; mAb, monoclonal Antibody; MDA, Malondialdehyde; MDSC, Myeloid-Derived Suppressor Cell(s); MHCII, Major Histocompatibility Complex Class II; MOG, Myelin Oligodendrocyte Glycoprotein; MS, Multiple Sclerosis; oxLDL, oxidized LDL; PFA, Paraformaldehyde; PTX, Pertussis Toxin; REMID, Repetitive Microdose Delivery; rIL, recombinant Interleukin; sc, subcutaneous; SPF, Specific-Pathogen-Free; Th, T helper; TE, Trolox Equivalents; TNFα, Tumor Necrosis Factor alpha; tolDC, tolerogenic Dendritic Cell(s); Tregs, Regulatory T Cells; VitD, Vitamin D3; WT, Wild Type.
