# Supplementary material for: IL-10-Dependent Amelioration of Chronic Inflammatory Disease by Microdose Subcutaneous Delivery of a Prototypic Immunoregulatory Small Molecule
Source: Front Immunol. 2021 Jul 8;12:708955. doi: 10.3389/fimmu.2021.708955 (PMC8297659; doi:10.3389/fimmu.2021.708955)
Supplement: Supplementary file 1 [file DataSheet_1.pdf]

Supplementary Material

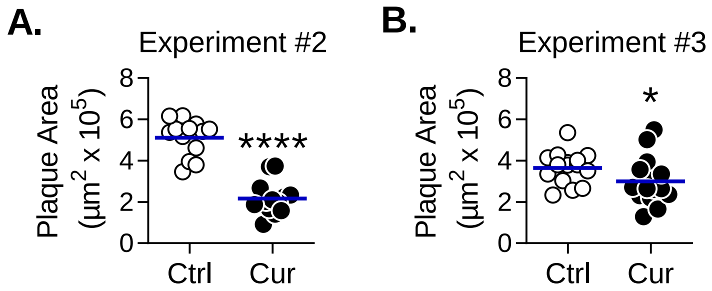

Supplementary Figure S1. Atheroprotective effects of REMID curcumin were confirmed in repetition experiments. ApoE-/- mice were treated as described in **Figure 1A** and cryosections from the aortic root were stained with H&E for plaque area assessment. **(A-B)** Curcumin-mediated atheroprotection was corroborated in two additional independent experiments. The areas of the atheromatous lesions were calculated with an image analysis software (see Materials and Methods). Each point represents the average area per mouse (7-9 sections/mouse), and bars represent the mean of 13-15. *P<0.05, ****P<0.0001 (Mann-Whitney *U* test).

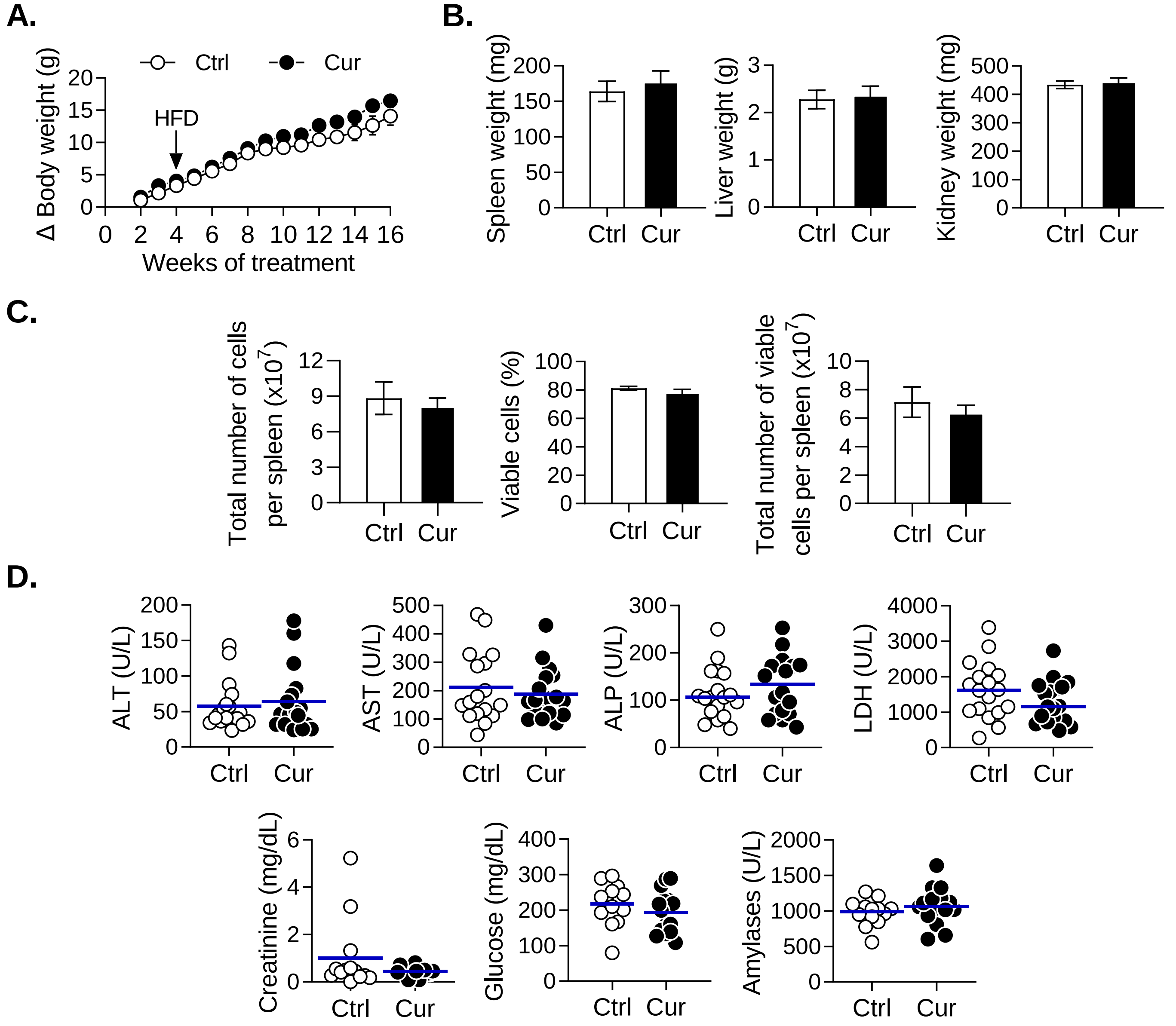

Supplementary Figure S2. The effects of REMID curcumin on body and organ weight, spleen cell counts and viability, and biochemical markers of liver, kidney, and pancreatic function. ApoE$^{-/-}$ mice were treated with curcumin or vehicle as shown in **Figure 1A**. **(A)** Changes of body weight were recorded weekly. Data is presented as the mean ± SEM. **(B)** Spleen, liver and kidneys were also weighted after animal euthanasia. **(C)** Determination of the total number of cells (left), viability (center) and total number of viable splenic cells (right) at the end of the experiment. **(B-C)** Data is presented as bars (mean ± SEM). **(D)** The levels of alanine aminotransferase (ALT), aspartate aminotransferase (AST), alkaline phosphatase (ALP), lactate dehydrogenase (LDH), creatinine, glucose and amylases were determined also at the end of the experiment. Each dot represents an individual animal and the bar the mean of the group. **(A)** Two-way ANOVA for repeated measures with Bonferroni's post-test and **(B-D)** Mann-Whitney U test showed no statistically significant differences between Cur and Ctrl groups for all measurements. Results in **(A-C)** are representative of two independent experiments (n=14-15 animals per group), and in **(D)** are pooled from two independent experiments. The arrow in **(A)** indicates the time of shift from chow to HFD diet.

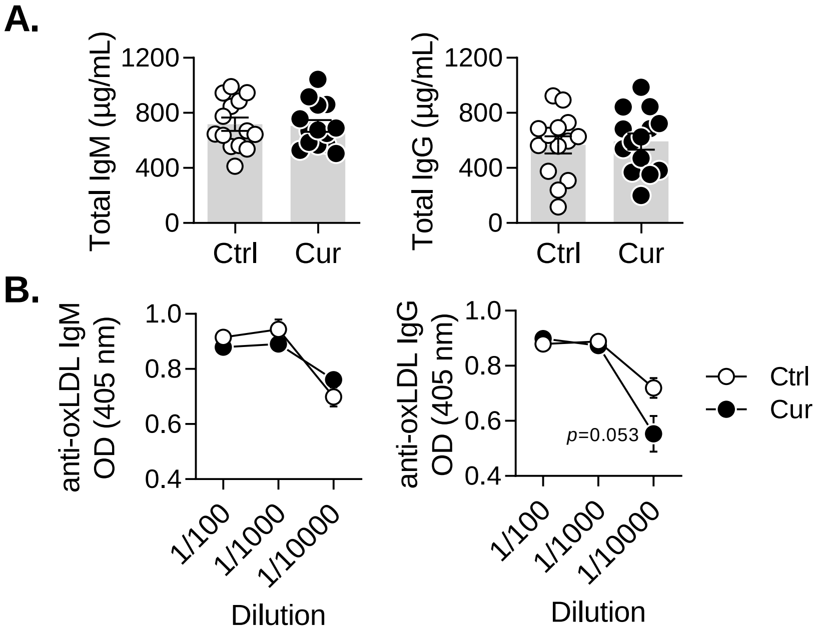

Supplementary Figure S3. No effect of sc low dose curcumin treatment on the circulating total and oxLDL-specific IgM and IgG antibody levels in dyslipidemic ApoE$^{-/-}$ mice. Serum samples from the ApoE$^{-/-}$ mice described in **Figure 1** were obtained at the end of the experiment to quantify **(A)** total and **(B)** oxLDL-specific IgM and IgG antibodies by ELISA. Representative results of two independent experiments are shown. Results are presented as the mean ± SEM; dots in **(A)** represent individual animals. n=10-15 mice per group. Mann-Whitney U test evidenced no significant differences between the Cur and Ctrl groups.

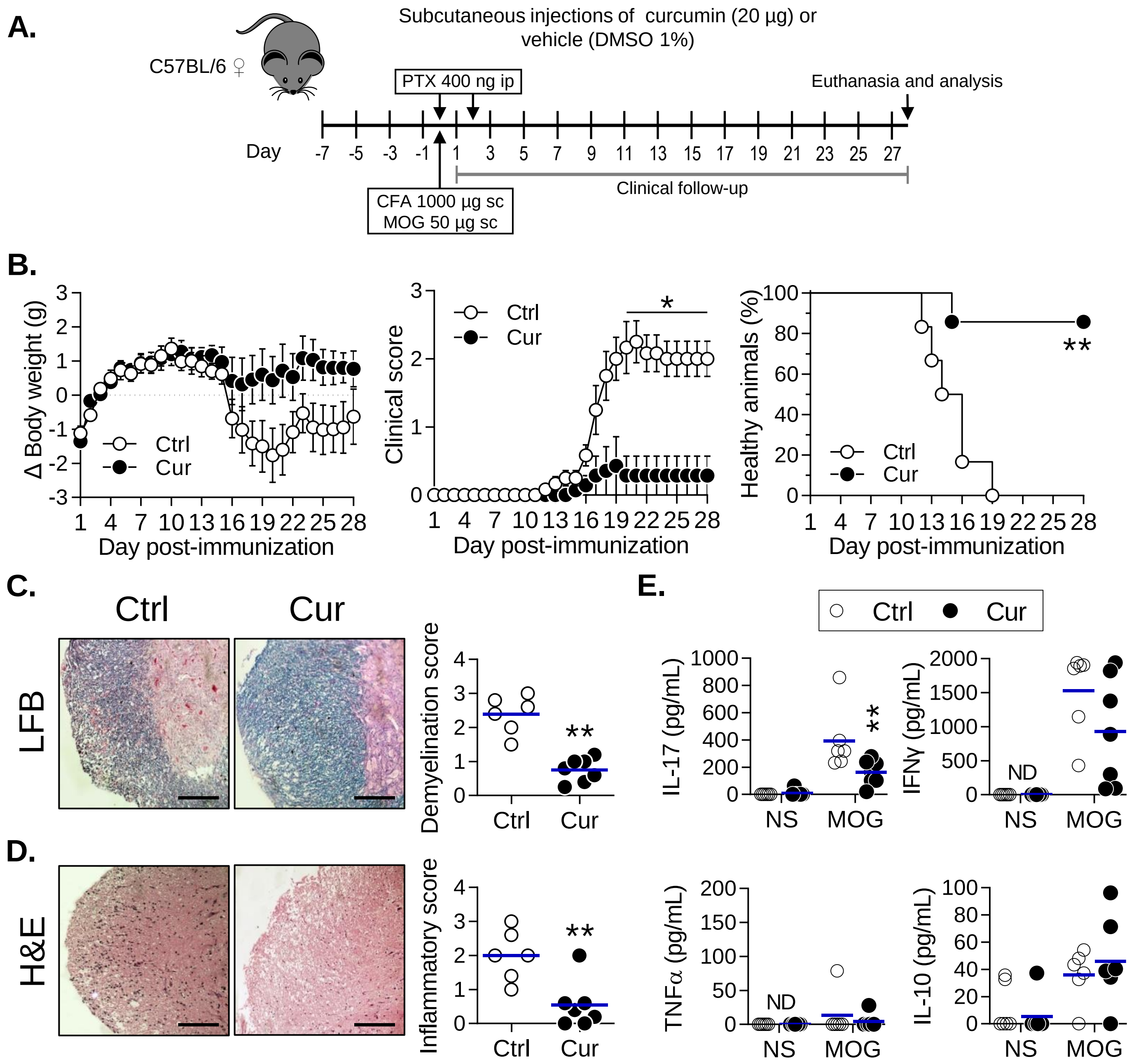

Supplementary Figure S4. A repetition experiment with sc REMID curcumin in the EAE model evidences reproducible neuroprotection. (A) A similar experiment to that depicted in **Figure 4A** was set with the only difference that the curcumin treatment and follow-up was extended to 28 days. Mice were euthanized and samples were collected at the end of the experiment (day 28). **(B)** Changes in body weight (left), clinical score (center) and percentage of healthy animals (right) are presented as the mean ± SEM (left and center) and as a Kaplan-Meier curve (right) from 6-7 mice/group. **(C-D)** Representative microphotographs from lumbar cord sections stained with **(C)** LFB and **(D)** H&E and the respective plots reporting demyelination and inflammatory scores in individual mice are presented. Each point is one animal and bars represent the mean of the group. Scale bar=25 µm. **(E)** Cytokine production response of MOG peptide-stimulated splenocytes is also shown. Non-stimulated (NS) control wells were also included. Results are plotted as individual animals and mean bars (mean ± SEM; n=6-7). *P<0.05; **P<0.01. Statistical tests were: **(B,** left and center**)** Two-way ANOVA for repeated measures with Bonferroni's post-test, **(B,** right**)** Mantel-Cox test and **(C-E)** Mann-Whitney U test.

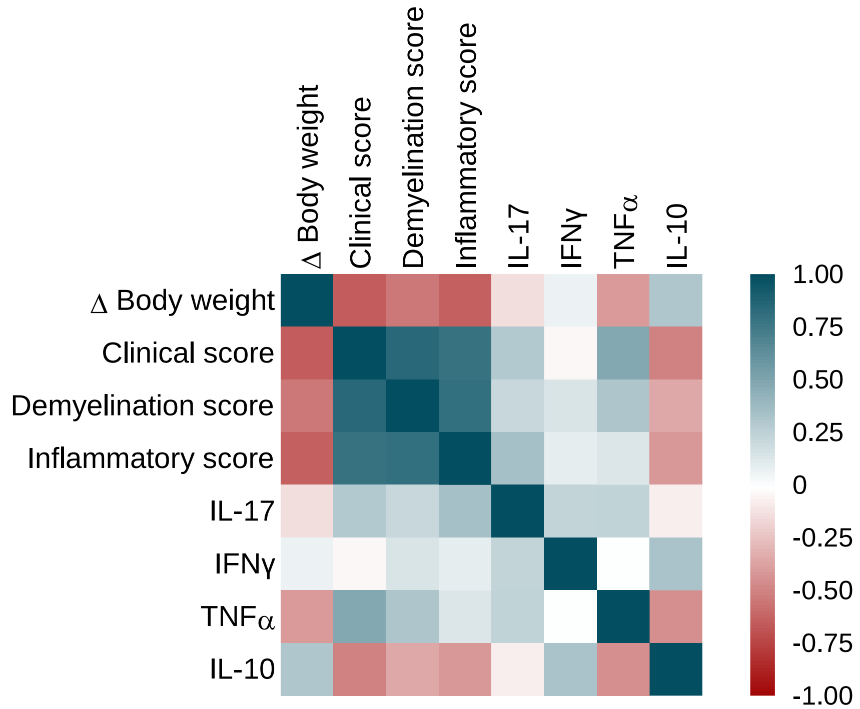

IL-10 vs.	Spearman r	*p* value
Clinical score	-0.496	0.002
Demyelination score	-0.343	0.044
Inflammatory score	-0.406	0.015
TNFα	-0.440	0.008

Supplementary Figure S5. A Spearman correlation matrix from EAE experiments showing that IL-10 induction correlates with disease amelioration. The relationships among eight measured parameters from EAE experiments showed in **Figure 4** and **Supplementary Figure S4** were estimated using Spearman rank correlation analysis. A heatmap of color intensity showing the magnitude of correlation is presented (blue: positive correlation, and red: negative correlation). This analysis confirmed a moderate but significant correlation between IL-10 production and clinical, histopathological, and inflammatory parameters (table).

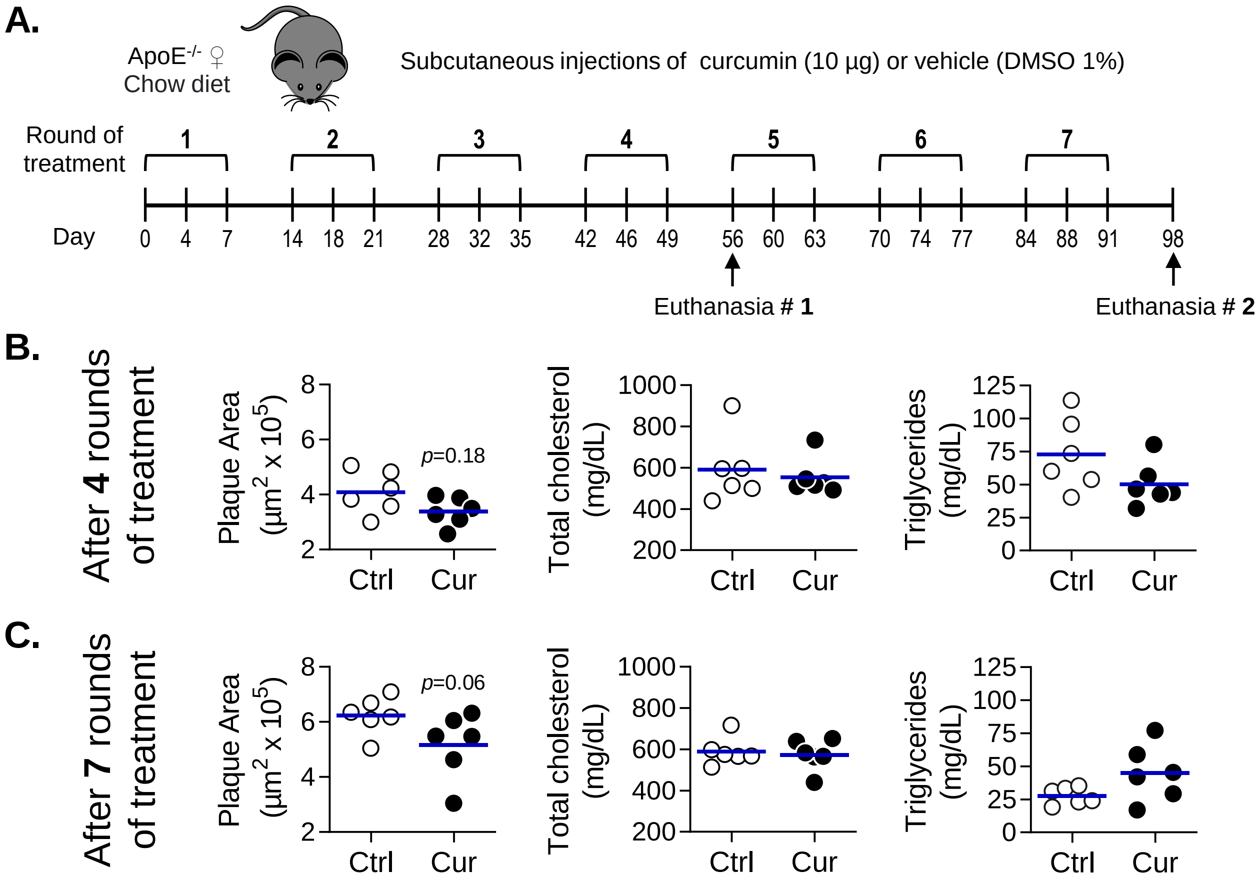

Supplementary Figure S6. Atheroprotective effect of microdose curcumin in female ApoE-/- mice fed a chow diet. (A) Dyslipidemic chow-diet-fed female ApoE-/- mice were treated three times a week (1 round of treatment), with subcutaneous injections of curcumin (Cur, 10 µg) or vehicle (Ctrl, DMSO 1 %) for 4 or 7 rounds every other week, euthanasia #1 and euthanasia #2, respectively. **(B-C)** Cryosections from the aortic root were stained with H&E to quantify plaque area (*left*) and serum samples were used to quantify total cholesterol (*center*) and triglycerides (*right*). Note that an increasing tendency to improve plaque area, but not serum lipids, is observed when treatment of mice is extended from 4 to 7 rounds. n=6 mice/group. Mann-Whitney *U* test.

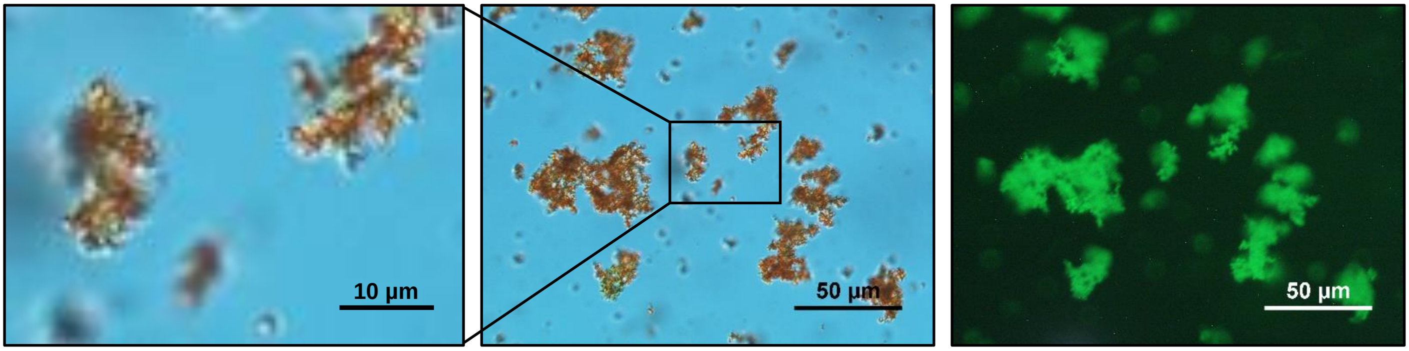

Supplementary Figure S7. The formulation used for atherosclerosis and EAE experiments forms curcumin aggregates. Curcumin dispersions were made in PBS containing 1% DMSO. Briefly, 20 mg of curcumin were dissolved in 1 mL DMSO and 10 µL of this solution were dispersed in 990 µL PBS 1X to finally obtain a suspension of 0.02% curcumin/1% DMSO that was used for the *in vivo* experiments. The preparation was mounted on a Nikon Eclipse Ti optical microscope and photographs with differential interference contrast (DIC, left and center) and fluorescence (right) were taken. Note that the curcumin/DMSO/PBS preparation prompted the aggregation of curcumin to form a colloidal suspension with heterogeneous particle size (from submicrometric up to 50 µm).
